# Identification of an Antagonistic Probiotic Combination Protecting Ornate Spiny Lobster (*Panulirus ornatus*) Larvae against *Vibrio owensii* Infection

**DOI:** 10.1371/journal.pone.0039667

**Published:** 2012-07-05

**Authors:** Evan F. Goulden, Michael R. Hall, Lily L. Pereg, Lone Høj

**Affiliations:** 1 Australian Institute of Marine Science, Townsville, Queensland, Australia; 2 Research Centre for Molecular Biology, School of Science and Technology, University of New England, Armidale, New South Wales, Australia; Auburn University, United States of America

## Abstract

*Vibrio owensii* DY05 is a serious pathogen causing epizootics in the larviculture of ornate spiny lobster *Panulirus ornatus*. In the present study a multi-tiered probiotic screening strategy was used to identify a probiotic combination capable of protecting *P. ornatus* larvae (phyllosomas) from experimental *V. owensii* DY05 infection. From a pool of more than 500 marine bacterial isolates, 91 showed definitive *in vitro* antagonistic activity towards the pathogen. Antagonistic candidates were shortlisted based on phylogeny, strength of antagonistic activity, and isolate origin. Miniaturized assays used a green fluorescent protein labelled transconjugant of *V. owensii* DY05 to assess pathogen growth and biofilm formation in the presence of shortlisted candidates. This approach enabled rapid processing and selection of candidates to be tested in a phyllosoma infection model. When used in combination, strains *Vibrio* sp. PP05 and *Pseudoalteromonas* sp. PP107 significantly and reproducibly protected *P. ornatus* phyllosomas during vectored challenge with *V. owensii* DY05, with survival not differing significantly from unchallenged controls. The present study has shown the value of multispecies probiotic treatment and demonstrated that natural microbial communities associated with wild phyllosomas and zooplankton prey support antagonistic bacteria capable of *in vivo* suppression of a pathogen causing epizootics in phyllosoma culture systems.

## Introduction

The ornate spiny lobster (*Panulirus ornatus*) is considered a prospective aquaculture species based on encouraging grow-out potential [1] and lucrative market value [2]. However, closed-life cycle production of *P. ornatus* is currently commercially unviable due to restricted production of postlarvae resulting from nutritional deficits and bacterial disease during their 4–6 month long larval phase [3]–[7]. *Vibrio owensii* is an emerging pathogen, with the type strain DY05 demonstrated as the etiological agent of a disease causing mass mortalities of cultured *P. ornatus* larvae (phyllosomas) [7], [8]. The pathogen can be transmitted through live feed vectors (*Artemia*) and proliferates in the phyllosoma hepatopancreas (midgut gland), causing extensive tissue necrosis and eventually major systemic infection [7].

In view of the global antibiotic resistance crisis [9] there is considerable interest in developing sustainable biocontrol methods such as probiotics for disease management in aquaculture [10]. The search for probionts is based on screening for beneficial microbial attributes such as antagonism, predation, anti-virulence, competition, attachment to host surfaces, and immunostimulation [10]–[14]. We have previously shown that the planktonic form is central to vectored transmission of *V. owensii* DY05 [7], hence it was pertinent to investigate the ability of probiotic candidates to inhibit planktonic growth. Moreover, since biofilms are refuges for pathogens in aquaculture systems [15], [16] and pathogen biofilms on natural tissues are inherently tolerant to conventional antimicrobial therapies [17], we also wanted to investigate the ability of probiotic candidates to inhibit biofilm formation under conditions of exclusion, competition and displacement.

Here we present a multi-tiered screening strategy for probiotic candidates, which ultimately led to the identification of a two-strain combination providing efficient protection of phyllosomas against experimental *V. owensii* DY05 infection. Initially, a shortlist of probiotic candidates was generated from a large pool of bacteria showing *in vitro* antagonism towards *V. owensii* DY05. Two additional *in vitro* screens were developed using a green fluorescent protein (GFP)-transconjugant of the pathogen to assess its planktonic growth and biofilm formation in the presence of shortlisted candidates. Subsequently, promising candidates were assessed for inherent virulence and protective benefit *in vivo* using a *P. ornatus* phyllosoma experimental infection model.

## Materials and Methods

### Replica Plate Assay

Wild *Panulirus* spp. phyllosomas and putative zooplankton prey items (not endangered or protected) were collected at Osprey Reef (Coral Sea, Australia; 13° 56′S, to 14° 03′ S and 144° 26′ E to 146° 48′ E) between 24 May-9 June 2008. No specific permits were required as the sites were located in the Australian Exclusive Economic Zone outside the Great Barrier Reef Marine Park and were not protected. Capture was achieved using a modified Isaac-Kidd mid-water trawl net according to Smith et al. [3]. Briefly, animals were washed 3× in 0.22 µm filtered artificial sea water (ASW; Instant Ocean®) to remove debris and loosely attached epibionts, homogenised in ASW, and spread plated on minimal marine agar (MMA; 0.3% casamino acids; 0.4% glucose; 1% bacteriological agar in 1 L ASW), modified from Hjelm et al. [18]. After incubation at ambient temperature (24°C) for 24–48 h, MMA plates with <300 colonies were replica plated [18] onto MMA seeded with 10 µL mL^−1^ of *V. owensii* DY05 grown overnight (24°C, 170 rpm) in marine broth 2216 (MB; BD). Replica plates were incubated for 72 h (24°C) and inspected for inhibition zones signifying antagonistic activity against *V. owensii* DY05. Antagonistic colonies were picked and cultured to purity on MMA, re-cultured in MB overnight (28°C, 170 rpm), and cryopreserved in 30% (v/v) glycerol (−80°C).

### Well-diffusion Agar Assay (WDAA)

Antagonistic isolates recovered from replica plates and the Australian Institute of Marine Science (AIMS) culture collection were tested for growth-inhibitory activity against *V. owensii* DY05 in a well diffusion agar assay (WDAA). In brief, the pathogen was seeded into molten MMA as outlined above. Following solidification, wells (diameter 5 mm) were cut into the agar and loaded with 40 µL of dense cultures (1–3 day old) of test isolates grown in MB (28°C, 170 rpm). Plates were incubated (28°C) and observed every 24 h for 72 h for inhibition zones. *Phaeobacter* (formerly *Roseobacter*) strain 27-4 was used as a positive antagonistic control on each plate because of its broad spectrum inhibitory activity against *Vibrio* pathogens [18]–[20]. Antagonism was classified according to the size of the inhibition zones as low (5–10 mm), moderate (11–20 mm) or strong (≥21 mm).

### Phylogenetic Identification

Colony PCR was performed on antagonistic isolates with universal primers 27F and 1492R [21] under standard conditions. PCR products were purified and sequenced using 27F (all strains) and 1492R (16 shortlisted candidates) as sequencing primers by Macrogen (Seoul, Korea). Sequences were edited with Sequencher 5.0 software (GeneCodes Corporation) and analysed using the BLAST algorithm (http://www.ncbi.nlm.nih.gov/BLAST/) to determine nucleotide-nucleotide similarity with sequences in the nr/nt database. Isolates were grouped according to phylogenetic relatedness by partial 16S rRNA gene sequence alignment using MEGA4 [22]. For the 16 shortlisted candidates sequences were submitted to GenBank under accession numbers JX075050–JX075065.

### Inoculum Preparation

A GFP-labelled transconjugant of *V. owensii* DY05 (DY05[GFP]) was used as a proxy for pathogen growth and attachment in microgrowth co-culture and multispecies biofilm assays, respectively (described below). DY05[GFP] stably expresses the GFP, and does not differ in growth profile or virulence towards stage 1 *P. ornatus* phyllosomas compared to wild type *V. owensii* DY05 [7].

DY05[GFP] was cultured on LB20 agar plates (5 g L^−1^ yeast extract; 10 g L^−1^ neutralised peptone; 20 g L^−1^ NaCl; 15 g L^−1^ agar) supplemented with 40 µg mL^−1^ kanamycin and 50 µg mL^−1^ colistin. Probiotic candidates and wild type *V. owensii* DY05 were cultured on marine agar 2216 (MA) at 28°C for 24 h. For each strain, colony material was suspended in 2 mL phosphate buffered saline (PBS: 8 g L^−1^ NaCl; 0.2 g L^−1^ KCl; 1.44 g L^−1^ Na_2_HPO_4_; 0.24 g L^−1^ KH_2_PO_4_; pH 7.2) and absorbance adjusted to OD_600 nm_ 0.1 (Nanodrop ND1000). The corresponding total viable counts (expressed as CFU mL^−1^) were determined for each strain in triplicate initial experiments by spiral plating (Eddy Jet; IUL) on MA and enumeration by an automatic colony counter (Flash and Grow v1.2; IUL). This information was used to calculate the volume of each OD_600 nm_ 0.1 suspension needed to achieve desired starting concentrations for the assays described below.

### Microgrowth Co-culture Assay

The relationship between fluorescence and CFU mL^−1^ of *V. owensii* DY05[GFP] monocultures was tested using the Pearson correlation coefficient. Triplicate samples were withdrawn from the microgrowth assay (described below) at 4 h intervals and there was a strong positive correlation (p<0.0001) between fluorescence and pathogen growth for the first 24 h (Figure S1). This showed that the fluorescence signal generated by *V. owensii* DY05[GFP] could be used to indirectly quantify pathogen growth during 24 h co-culture with probiotic candidates.

To assess the activity of planktonic candidates, MB was inoculated with PBS suspensions of *V. owensii* DY05[GFP] (initial concentration 1×10^3^ CFU mL^−1^) separately or in combination with PBS suspensions of candidate probiotics (final concentrations 1×10^3^, 1×10^5^, or 1×10^7^ CFU mL^−1^) in Nunc™ (NUN137101) black microwell plates (final volume 200 µL). Separate plates were used for each candidate-pathogen combination and all treatments were performed in hextuplicate well sets. Sterile milli-Q water (200 µL) was added to perimeter rows and columns to minimise evaporative loss and plate covers were treated with 0.1% Triton X-100 in 20% ethanol to smooth condensation [23]. Plates were sealed with parafilm and incubated for 24 h (28°C, 170 rpm). Growth of the GFP-tagged pathogen was monitored indirectly by measuring fluorescence (excitation/emission 485/520 nm) with a Wallac Victor^2^ 1420 multilabel counter. Fluorescence values were adjusted by subtracting the average autofluorescence generated from corresponding controls (wild type *V. owensii* DY05 monocultures or co-cultures).

The antagonistic activity of each strain was classified based on their ability to reduce the pathogen fluorescence signal after 24 h relative to the maximum signal reduction recorded for the respective assay. In this way, strain antagonistic activity was classified as low (<50% of max), moderate (50–75% of max), or strong (>75% of max).

### Biofilm Assays

Monostrain biofilm production by the pathogen and candidate probionts was quantified using a microwell crystal violet (CV) staining assay [24], modified so culture conditions were consistent with other microwell assays used in the present study (Protocol S1). Pathogen biofilm formation in the presence of probiotic candidates was tested under conditions of exclusion, competition, and displacement using a modified attachment assay [25]. The sensitivity of the assay was increased by extending the incubation time from 1 h to the time of maximum biofilm formation by the reporter strain (DY05[GFP]) as determined by the CV assay (t = 48 h and t = 72 h).

For each test, MB was inoculated with PBS suspensions of bacterial strains (each at initial concentration 1×10^7^ CFU mL^−1^) in Optical bottom Nunc™ (NUN165305) microwell plates (final well volume 200 µL). In all cases the time point for addition of DY05[GFP] was set as t = 0 and plates were incubated statically at 28°C until t = 48 h or t = 72 h. Specifically, for the exclusion assay, half volume MB was inoculated with probiotic candidates and incubated for 24 h prior to inoculation with DY05[GFP] (t = 0 h) and fresh MB. For the competition assay, MB was inoculated simultaneously with DY05[GFP] and probiotic candidates (t = 0 h). For the displacement assay, half volume MB was inoculated with DY05[GFP] (t = 0 h) and incubated for 24 h before inoculation with candidate probionts and fresh MB.

Separate plates were used for each interaction and incubation period and treatments were carried out in hextuplicate well sets. Plates were sealed and treated as described for microgrowth co-culture assay. After incubation, wells were washed 3× in 200 µL PBS to remove planktonic and nonadherent cells. To prevent dehydration, washed wells were loaded with PBS (200 µL). Biofilm attachment was measured as a function of fluorescence and fluorescence values adjusted as described above. Candidates which caused significant signal increase (Student t-test, p<0.05) at any time point were removed from the candidate pool as these were considered pathogen biofilm facilitators. From the remaining observations, the average signal decrease was calculated from the two time points and used to classify the antagonistic activity of each strain as described above.

### *In vivo* Protection against *V. owensii* DY05 Infection

Selected candidates were tested for inherent pathogenicity towards cultured stage 1 *P. ornatus* phyllosomas using an infection model described previously [7]. Briefly, phyllosomas were exposed to bacterial strains using live *Artemia* stage II (nauplii) as vectors. Formalin-disinfected nauplii were enriched with probiotic candidates through filter-feeding in tissue culture flasks (Sarstedt) for 2 h (initial concentration 1×10^7^ CFU mL^−1^). Positive pathogen control nauplii and negative control nauplii were treated similarly, except 1×10^6^ CFU mL^−1^ *V. owensii* DY05 or no bacteria were added, respectively. Apparently healthy *P. ornatus* phyllosomas, as assessed by photopositive response and active motility, were sourced from the AIMS larviculture facility [3], distributed to 12-well cell culture plates (1 larva well^−1^) and fed live enriched or non-enriched nauplii (t = 0 h). All treatments were performed in quintuplicate (*n* = 60) and survival was assessed every 24 h for 5 days.

The protective benefit of selected candidates was evaluated in two separate initial experiments using the same probiotic administration strategy (Strategy 1). In brief, stage 1 *P. ornatus* phyllosomas were fed nauplii enriched with probiotic candidates separately, or in combination (t = 0 h; each candidate at 1×10^7^ CFU mL^−1^). After 24 h, phyllosomas were challenged with nauplii enriched with 1×10^6^ CFU mL^−1^ *V. owensii* DY05 for 6 h, after which phyllosomas were transferred to new cell culture plates and again fed nauplii enriched with probiotic candidates (t = 30 h). Subsequently, in a third experiment the most promising candidates were tested using an alternative administration strategy (Strategy 2) which differed from strategy 1 by enriching nauplii with the pathogen in combination with probiotic candidates at t = 24 h (pathogen at 1×10^6^ CFU mL^−1^; each probiont at 1×10^7^ CFU mL^−1^). The most promising treatment from the latter experiment was replicated twice to validate observations. All experiments included a negative control (non-enriched nauplii) and a positive pathogen control (nauplii enriched in *V. owensii* DY05 at t = 24 h; non-enriched nauplii at t = 0 and t = 30 h). All treatments were performed in quintuplicate (*n* = 60) and survival was assessed every 24 h for 5 days.

### Statistical Analysis

Differences between survival curves in the experimental infection models were determined using the product-limit (Kaplan-Meier) estimator and confirmed with an ANOVA. A post hoc Dunnett’s test was used to compare treatments to the defined control groups. All statistical analyses were performed using the statistical software package JMP®7 (SAS) standardised at significance level α = 0.05.

## Results

### Antagonistic Bacteria

The WDAA confirmed 62 of 149 isolates recovered from replica plating and 29 of 356 culture collection isolates as antagonistic towards *V. owensii* DY05. The majority of confirmed antagonistic isolates belonged to the genera *Pseudoalteromonas* (66 isolates; 1 pigmented and 1 non-pigmented phylotype) and *Vibrio* (16 isolates; 4 phylotypes). The remainder belonged to the *Bacteroidetes* phylum (2 isolates; 1 phylotype), and the genera *Ruegeria* (3 isolates; 2 phylotypes), *Bacillus* (2 isolates; 2 phylotypes), *Psychrobacter* (1 isolate) and *Acinetobacter* (1 isolate).

To select antagonistic strains likely capable of interaction with phyllosoma hosts, representatives were shortlisted based on their phylogenetic identity, the strength of their *in vitro* antagonism in WDAA, and their environmental origin, with preferences given towards natural prey-derived isolates and bacteria associated with *P. ornatus* phyllosomas or their environments (natural or artificial). Strains that were closely related to known human pathogens or were difficult to keep in pure culture were excluded from further analysis. In this way, the pool of probiotic candidates was reduced to 16 isolates for further *in vitro* screening (Table S1).

### Co-culture Assay

**Figure 1:**
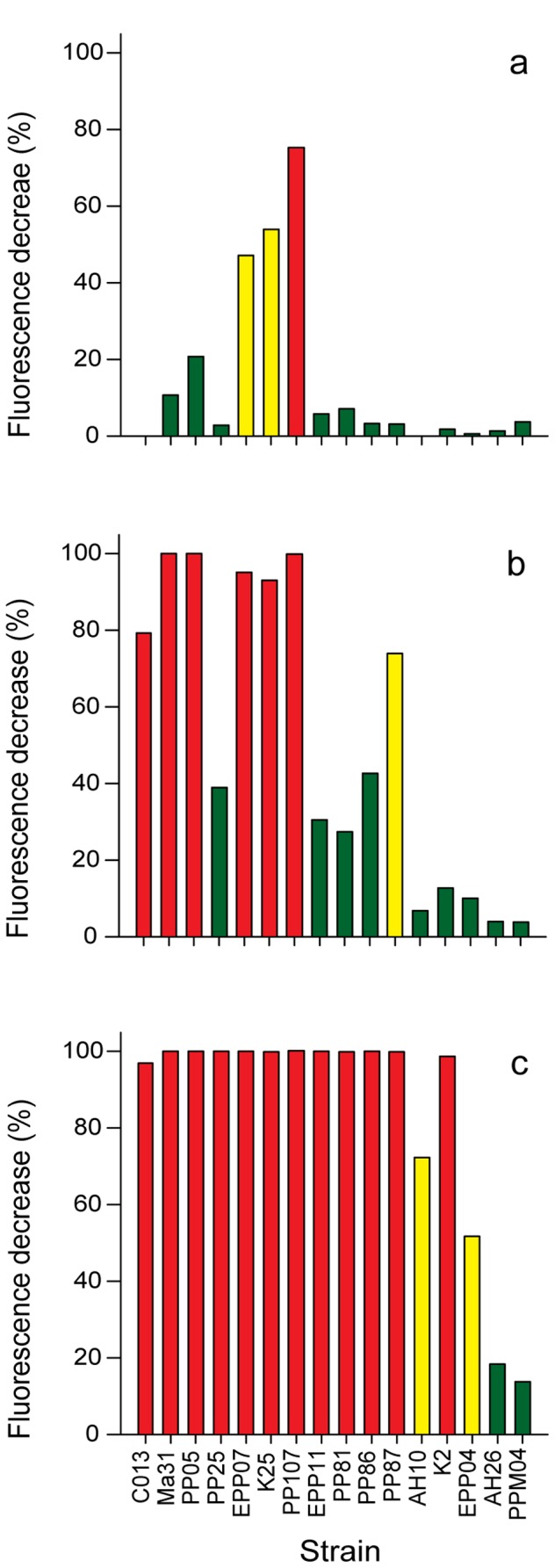
Microgrowth co-culture assay. Inhibitory effect probiotic candidates on pathogen growth determined after 24 h co-culture, using fluorescence expressed by *V. owensii* DY05[GFP] as a proxy for its planktonic growth. The initial pathogen concentration was 1×10^3^ CFU mL^−1^, while initial probiont concentrations were (a) 1×10^3^ CFU mL^−1^, (b) 1×10^5^ CFU mL^−1^, or (c) 1×10^7^ CFU mL^−1^. Green: low activity (<50% of max); Yellow: moderate activity (50–75% of max); Red: strong activity (>75% of max).

Probiotic candidates were co-cultured with *V. owensii* DY05[GFP] at three different starting concentrations (Figure 1). At equal starting concentrations (1×10^3^ CFU mL^−1^), the probiotic candidates had little or no inhibitory effect on pathogen growth, except for the three pigmented *Pseudoalteromonas* strains (EPP07, K25 and PP107), which demonstrated moderate to strong activity. At higher initial concentration (1×10^5^ CFU mL^−1^), the candidates generally had increased inhibitory effect, with strong inhibition of pathogen growth observed for the pigmented *Pseudoalteromonas* strains (EPP07, K25 and PP107) and three of the four *Vibrio* strains (C013, Ma31, and PP05). At the highest initial concentration (1×10^7^ CFU mL^−1^), all candidates belonging to the *Pseudoalteromonas* and *Vibrio* genera, and *Ruegeria* strain K2 caused strong growth inhibition, in some instances resulting in total elimination of fluorescent signals. Irrespective of initial concentration, the *Bacteroidetes* strains (AH26 and PPM04) had minor impact on pathogen growth during co-culture.

### Monostrain Biofilm Production

**Figure 2:**
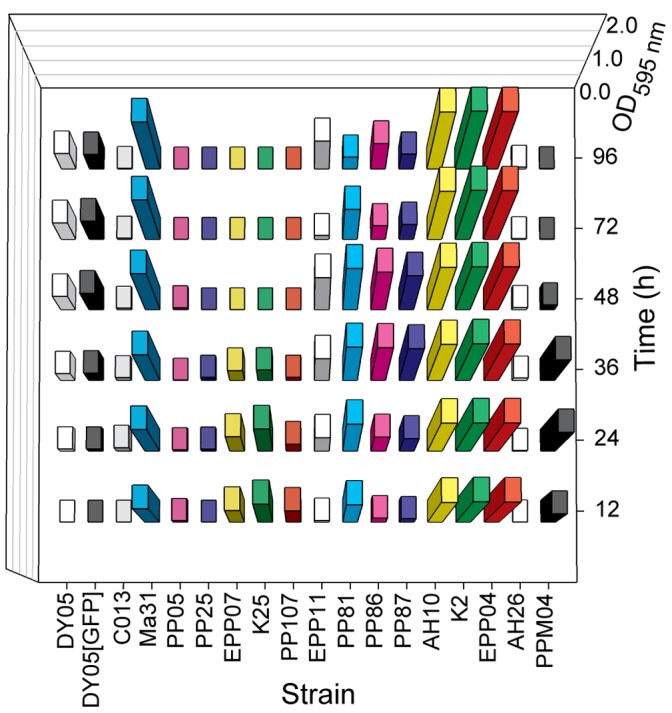
Monostrain biofilm formation. A crystal violet-microwell assay was used to assess monospecies biofilm formation of *V. owensii* DY05, *V. owensii* DY05[GFP] and probiotic candidate isolates belonging to *Vibrio* (C013, Ma31, PP05, and PP25), *Pseudoalteromonas* (EPP07, K25, PP107, EPP11, PP81, PP86, and PP87), *Ruegeria* (AH10, K2, and EPP04), and *Bacteroidetes* (AH26 and PPM04).

A microwell crystal violet assay was used to study monostrain biofilm production (Figure 2). Attachment of *V. owensii* DY05 and DY05[GFP] was detected after 24 h but maximum biofilm density was achieved between 48 and 72 h prior to dispersal. Pigmented *Pseudoalteromonas* strains (EPP07, K25 and PP107) rapidly formed dense biofilms which dispersed after 48 h. Non-pigmented *Pseudoalteromonas* strains (EPP11, PP86, and PP87) were strong biofilm formers reaching maximum density between 36 and 48 h. Strain PP81 differed from other non-pigmented pseudoalteromonads by rapidly forming and maintaining biofilm density for 48 h until sloughing. *Vibrio* strains generally produced low density biofilms, with exception of Ma31 which formed a dense and stable biofilm after 12 h. The *Ruegeria* isolates (AH10, K2, and EPP04) were strong and stable biofilm formers. *Bacteroidetes* strain PPM04 rapidly formed and maintained a dense biofilm between 12 and 36 h before dispersal, while *Bacteroidetes* strain AH26 was a weak biofilm producer.

### Multistrain Biofilm Assays

**Figure 3:**
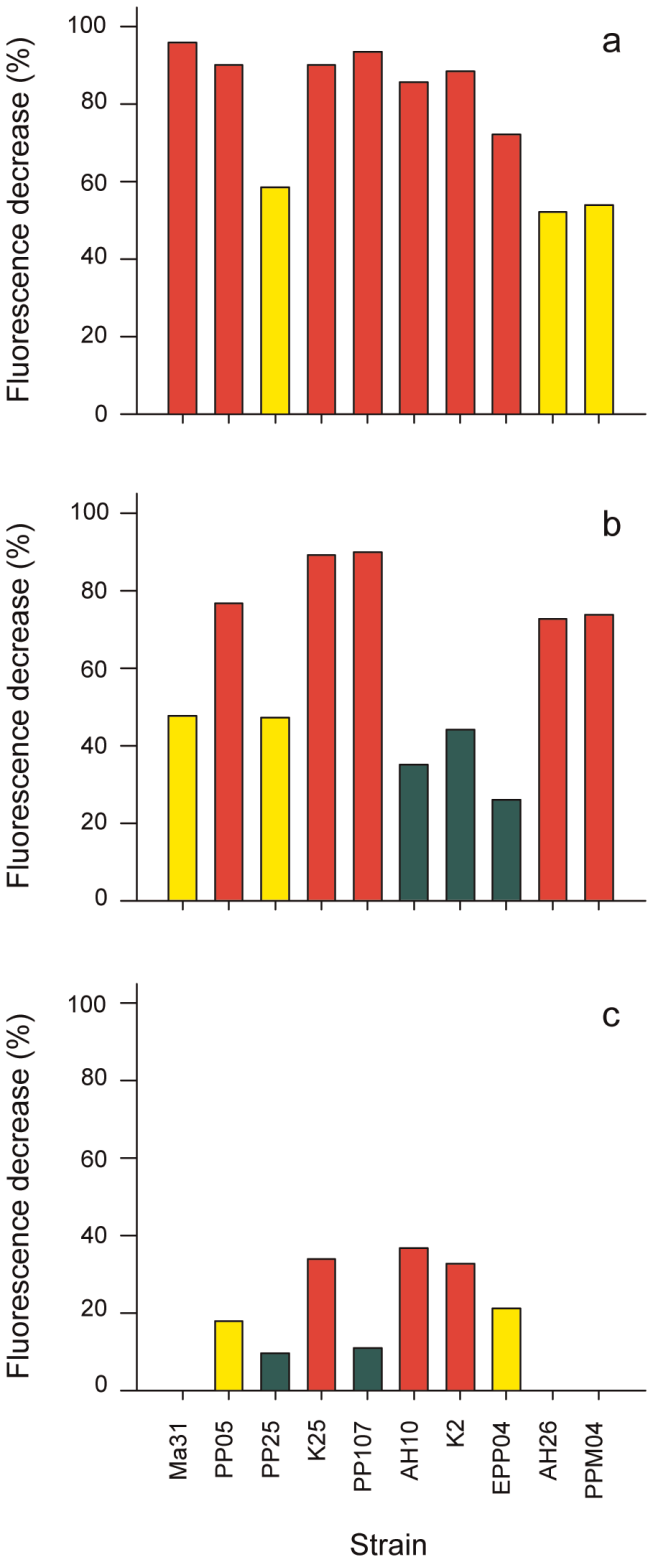
Multistrain biofilm interactions. Inhibitory effect of probiotic candidates on pathogen biofilm formation under conditions of (a) exclusion, (b) competition and (c) displacement using fluorescence expressed by *V. owensii* DY05[GFP] as a proxy for pathogen attachment. Strains that appeared to facilitate pathogen biofilm formation are not presented. Columns represent average values from two time points (t = 48 h and t = 72 h). Green: low activity (<50% of max); Yellow: moderate activity (50–75% of max); Red: strong activity (>75% of max).

A multistrain biofilm assay was used to investigate pathogen biofilm formation in the presence of probiotic candidates under conditions of exclusion, competition and displacement (Figure 3). All non-pigmented *Pseudoalteromonas* strains (EPP11, PP81, PP86, and PP87), the pigmented *Pseudoalteromonas* strain EPP07, and *Vibrio* strain C013 caused significant increases (Student’s t test p<0.05) in fluorescence signals in at least one multistrain interaction, and hence were regarded as biofilm facilitators and eliminated from the candidate pool. In terms of percentage signal reduction, the remaining 10 candidates were more successful at inhibiting pathogen biofilm by exclusion (52–96%; Figure 3a), followed by competition (26–90%; Figure 3b) and displacement (0–37%; Figure 3c). Under conditions of exclusion, the pigmented *Pseudoalteromonas* strains K25 and PP107, *Vibrio* strains Ma31 and PP05, and all *Ruegeria* isolates (AH10, K2 and EPP04) demonstrated strong inhibition. In the competition assay, strong inhibitory activity was shown by two pigmented *Pseudoalteromonas* strains (K25 and PP107), *Vibrio* strain PP05 and the *Bacteroidetes* strains (AH26 and PPM04), and weakest activity exhibited by the *Ruegeria* candidates AH10, K2 and EPP04. During conditions of displacement, the strongest inhibitory activity was exhibited by two *Ruegeria* candidates (AH10 and K2) and pigmented *Pseudoalteromonas* strain K25.

### Pathogenicity Testing and *in vivo* Protective Benefit

The selection of candidates showing strong antagonistic activity under all tested conditions was regarded as the most promising strategy for successful inhibition of the pathogen during *in vivo* conditions. Based on *in vitro* screening results the strains *Vibrio* sp. PP05, *Pseudoalteromonas* sp. PP107, and *Pseudoalteromonas* sp. K25 were selected based on their overall superior performance in the well diffusion, microgrowth and biofilm assays. In addition, the best performing *Roseobacter* clade isolate (*Ruegeria* sp. K2) was included since many strains belonging to this group of bacteria have elicited promising probiotic effects for other aquaculture species.

**Figure 4:**
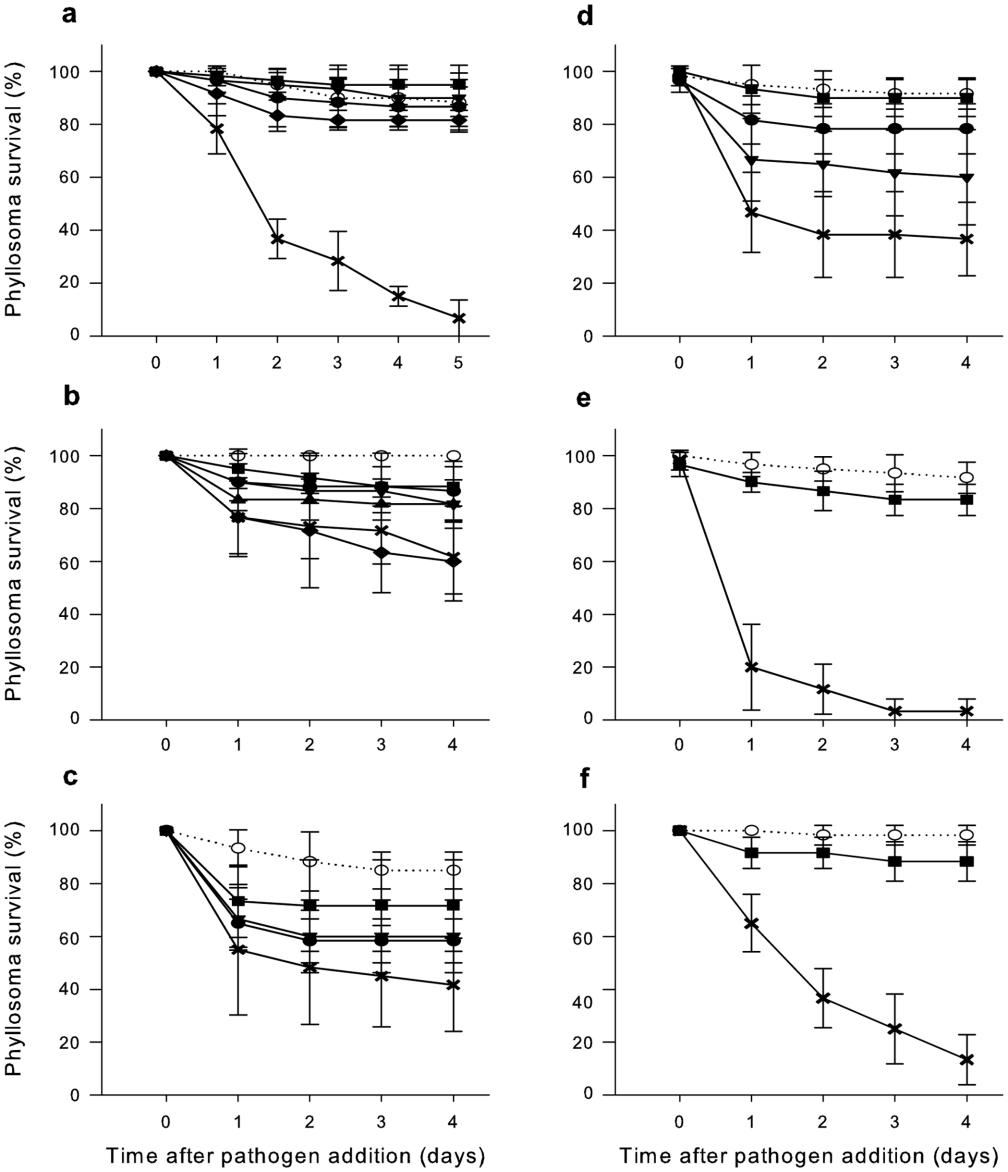
Pathogenicity testing and protective benefit of probiotic candidates on pathogen challenged *P. ornatus* phyllosomas. (a) Pathogenicity testing of probiotic candidates. *Vibrio* sp. PP05 (•), *Pseudoalteromonas* sp. PP107 (▪), *Pseudoalteromonas* sp. K25 (▾), or *Ruegeria* sp. K2 (♦), unchallenged control (○), *V. owensii* DY05 pathogen control (×). (b) Protective benefit of probiotic candidates towards *V. owensii* DY05 challenged phyllosomas using Administration Strategy 1. PP05 (•), PP107 (▪), K25 (▾), K2 (♦), a mixture of the four candidates (▴), unchallenged control (○), pathogen control (×). (c) Protective benefit of probiotic candidates towards *V. owensii* DY05 challenged phyllosomas using Administration Strategy 1. PP05 (•), PP107 (▾), PP05+ PP107 (▪), unchallenged control (○), pathogen control (×). (d) Protective benefit of probiotic candidates towards *V. owensii* DY05 challenged phyllosomas using Administration Strategy 2. PP05 (•), PP107 (▾), PP05+ PP107 (▪), unchallenged control (○), pathogen control (×). (e-f) Replicate experiments showing protective benefit of probiotic candidates towards *V. owensii* DY05 challenged phyllosomas using Administration Strategy 2. PP05+ PP107 (▪), unchallenged control (○), pathogen control (×). Phyllosoma survival expressed as Means ± SD.

An initial experiment tested if the probiotic candidates themselves were pathogenic to phyllosomas. Vectored challenge with PP05, PP107, K25 or K2 (Dunnett’s test p>0.05) did not alter survival of *P. ornatus* phyllosomas (stage 1) relative to the unchallenged control (Figure 4a) and no anomalous phototactic responses or swimming behaviours were noted, indicating the candidates were non-pathogenic. In comparison, vectored challenge with *V. owensii* DY05 (pathogen control) caused a significant increase (Dunnett’s test p<0.0001) in phyllosoma mortality (Figure 4a).

The *in vivo* protective potential of candidates was tested in an experimental setup where probiotic candidates were delivered to phyllosomas via *Artemia* nauplii before (t = 0 h) and after (t = 30 h) vectored challenge with the pathogen *V. owensii* DY05 for 6 h (t = 24–30 h) (Strategy 1). Phyllosoma survival was significantly enhanced for candidates PP05 and PP107 (Dunnett’s test p<0.05) compared to the pathogen control (Figure 4b). In contrast, treatment with K25, K2 or a mixture of the four candidates (Dunnett’s test p>0.05) did not significantly enhance survival relative to the pathogen control (Figure 4b). Based on these results, PP05 and PP107 were selected as the most promising candidates and their protective benefit singularly or in combination was further investigated. In the second experiment, survival of PP05 or PP107-treated phyllosomas did not significantly differ from the pathogen control (Dunnett’s test p>0.05) (Figure 4c). However, used in combination the probiotic candidates resulted in a significant (Dunnett’s test p<0.01) benefit, enhancing phyllosoma survival by 30% (Figure 4c).

When the administration strategy was altered so that probionts were present also during the pathogen challenge (t = 24–30 h) (Strategy 2), phyllosoma survival was enhanced by 23% for PP107 (Dunnett’s test p<0.01), by 42% for PP05 (Dunnett’s test p<0.0001), and by 53% for PP05/PP107 in combination (Dunnett’s test p<0.0001) relative to the pathogen control (Figure 4d). The experiment was repeated twice for PP05/PP107 in combination to validate observations (Figure 4e–f), and phyllosoma survival was significantly enhanced by 80% (Dunnett’s test p<0.0001) and 75% (Dunnett’s test p<0.0001) respectively, compared to the pathogen control (Figure 4e–f). It should be noted that survival of PP05/PP107-treated phyllosomas did not differ significantly (Dunnett’s test p>0.05) from non-challenged control phyllosomas in each of the three replicated experiments when nauplii were enriched simultaneously with pathogen and probionts (Figure 4d–f).

## Discussion

The present study has demonstrated that antagonistic bacteria recovered from natural prey items of *P. ornatus* phyllosomas were capable of protecting cultured phyllosomas from the serious hatchery pathogen *V. owensii* DY05. The used probiotic screening strategy targeted antagonistic activity by candidate strains in both planktonic and attached forms, resulting in the selection of a two strain combination (*Vibrio* sp. PP05 and *Pseudoalteromonas* sp. PP107) that conferred a substantial additive survival benefit to pathogen-challenged phyllosomas.

Antagonism is a widespread trait implicated in the competiveness and ecological success of many marine bacteria [26]–[29] and is thus considered an important attribute of aquaculture probionts. In the present study, the antagonistic bacteria most readily culturable from wild phyllosomas and zooplankton belonged to *Pseudoalteromonas* and *Vibrio*. Both genera are frequently recovered from the marine environment and aquaculture systems [18], [27]–[30] and are commonly associated with eukaryotic hosts [31], [32]. It is not known which antagonistic mechanisms were used by strains tested in the current study, however broad-spectrum anionic proteins and non-proteinaceous antibiotics produced by *Pseudoalteromonas* spp. [33], [34], and aliphatic hydroxyl ethers and andrimid antibiotics [30], [35] synthesised by *Vibrio* spp. are implicated in inhibition of aquatic vibrios.

Inhibition of planktonic *V. owensii* DY05 depended on both initial concentration and taxonomic grouping of the candidates. The results support previous studies showing that antagonists are generally required at higher concentrations than the pathogen for elimination [36]–[38]. Planktonic forms of all *Vibrio* and *Pseudoalteromonas* strains and the *Ruegeria* strain K2 strongly inhibited pathogen growth at the highest inoculum concentration. In contrast, the other strains (eg. *Bacteroidetes* and the other *Ruegeria* candidates) showed only moderate or low inhibition of planktonic pathogen growth despite showing antagonistic activity in well diffusion assays. This observation is consistent with several studies suggesting that free-living forms of marine bacteria may be less prone to producing antibacterials [26], [29]. Moreover, some compounds may only be bioactive during certain interactions. For example, Dheilly et al. [39] found anti-biofilm exoproducts of *Pseudoalteromonas* sp. 3J6 had no antibacterial properties against free-living *Paracoccus* and *Vibrio* strains.

Overall, the strongest biofilm inhibitory activity was seen in the exclusion assay, followed by the competition and displacement assays, respectively. This was probably due to the ability of bacteria such as *Pseudoalteromonas* and *Ruegeria* to rapidly form biofilms on the microwell surface and synthesise compounds with antifouling and antibacterial activity [20], [40], [41] that resist incoming pathogen propagules. The pigmented *Pseudoalteromonas* strains were the strongest inhibitors of pathogen attachment and pigmentation is known to be linked to production of bioactive molecules in this genus [40], [42]. Some *Vibrio* candidates (PP05 and PP25) were poor biofilm formers on the microwell surface yet were among the strongest inhibitors of pathogen attachment, suggesting inhibition was probably more related to the potency of the secreted compound rather than the biofilm biomass. Attached *Bacteroidetes* strains (AH26 and PPM04) were more successful than their planktonic conspecifics in outcompeting the pathogen, inferring an ecological preference for surface attachment and supporting a growing body of evidence that attached forms are more likely to exhibit antibacterial activity [29]. Reduced ability of probiotic candidates to displace pathogen biofilms could partly be related to the biofilm exopolymeric matrix trapping or slowing diffusion of antimicrobial compounds, leading to increased resistance [43], [44]. Biofilms may also tolerate antimicrobials through changes in genotypic pathways, including upregulation of genes encoding efflux pumps which facilitate the efflux of antimicrobials [45].

An experimental phyllosoma infection model was used to investigate if treatment with probiotic candidates could prevent or interrupt the infection cycle of *V. owensii* DY05 in *P. ornatus* phyllosoma. A pathogen exposure time of 6 h was selected, as previous studies using the phyllosoma infection model showed that *V. owensii* DY05 cells have entered the hepatopancreas at this time point [7]. However, as opportunistic carnivores [46], [47] some phyllosomas had not consumed all *Artemia* nauplii after 6 h, likely contributing to increased standard deviations relative to the robust and reproducible survival data produced in our previous study [7]. This can in part explain the discrepancies between the first two protection experiments (Figure 4b–c), where a significant protective benefit was recorded for separate treatments with *Vibrio* sp. PP05 or *Pseudoalteromonas* sp. PP107 in the first but not the second experiment.

An altered delivery strategy where the pathogen was added in combination with probiotic candidates during vectored transmission dramatically increased the protective benefit towards phyllosomas. Importantly, survival of phyllosomas receiving *Vibrio* sp. PP05 and *Pseudoalteromonas* sp. PP107 in combination did not differ significantly from non-treated controls across three replicated experiments (Figure 4d–f), and a reproducible survival enhancement (53–80%) was seen relative to pathogen controls. *Pseudoalteromonas* and *Vibrio* species have previously shown good potential as probiotics by enhancing survival of cultured invertebrates [33], [48], [49] and fish [50], [51] following challenge with pathogenic vibrios. However, horizontal gene transfer has contributed significantly to the evolution and dissemination of virulence genes in *Vibrio* genomes [52], so a certain amount of risk is involved in the selection of *Vibrio* probiotic strains. We consider the risk to be acceptable given the lack of evidence of probiotic vibrios acquiring virulence traits and the dramatically increased protective effect on phyllosomas when *Vibrio* sp. PP05 was included in the probiotic mixture. The possibility of transfer of virulence traits to PP05 does however exist, and this will have to be considered if disease outbreaks persist or re-emerge.

Multispecies probiotic applications have shown clear advantages over monospecies formulations in improving pathogen resistance also in previous studies [53]. At this stage it is not clear which mechanisms are responsible for the additive probiotic effects of PP05 and PP107 and this warrants further investigation.

### Conclusions

*Vibrio* sp. PP05 and *Pseudoalteromonas* sp. PP107 were prospected from a large pool of antagonistic candidates based on their ability to inhibit planktonic and attached forms of pathogenic *V. owensii* DY05. Used in combination, these bacteria significantly and reproducibly protected *P. ornatus* phyllosomas from experimental infection with *V. owensii* DY05. Thus, the use of miniaturised co-culture and biofilm assays enabled rapid processing of numerous candidates and selection of probiotic bacteria capable of promoting survival. The study showed that natural microbial communities of wild phyllosomas support antagonistic bacteria capable of suppressing pathogens originating from the larviculture ecosystem and affirmed natural prey items as reservoirs of beneficial microorganisms.

## Supporting Information

Figure S1 **Correlation between fluorescence and concentration (CFU mL^−1^) of** ***V. owensii*** **DY05[GFP] during 24 h monoculture growth.**
(DOCX)

Table S1 **Probiotic candidate shortlist.**
(DOCX)

Protocol S1 **Monostrain biofilm production assay.**
(DOCX)
