# Supplementary material for: Identification of an Antagonistic Probiotic Combination Protecting Ornate Spiny Lobster (Panulirus ornatus) Larvae against Vibrio owensii Infection
Source: PLoS One. 2012 Jul 5;7(7):e39667. doi: 10.1371/journal.pone.0039667 (PMC3390342; doi:10.1371/journal.pone.0039667)
Supplement: Table S1 — Probiotic candidate shortlist. (DOCX) [file pone.0039667.s002.docx]

**Table S1:** Probiotic candidate shortlist

| Taxonomic group | Strain | GenBank Accession no. | | Source | Antagonistic activity^b^ |
| --- | --- | --- | --- | --- | --- |
| *Vibrio* sp. (PT1) | C013 | JX075050 | Surface water, Coral Sea | | Low |
| *Vibrio* sp. (PT2) | Ma31 | JX075051 | Aged seawater (AIMS) | | Low |
| *Vibrio* sp. (PT3) | PP05 | JX075052 | Salp (*Salpida*) | | Moderate |
| *Vibrio* sp. (PT4) | PP25 | JX075053 | Salp (*Salpida*) | | Moderate |
| *Pseudoalteromonas* sp. (PT1)^c^ | EPP07 | JX075054 | *P. ornatus* phyllosoma, cultured | | Strong |
| *Pseudoalteromonas* sp. (PT1)^c^ | K25 | JX075055 | Larviculture water (AIMS) | | Strong |
| *Pseudoalteromonas* sp. (PT1)^c^ | PP107 | JX075056 | Arrow worm (*Chaetognatha*) | | Moderate |
| *Pseudoalteromonas* sp. (PT2) | EPP11 | JX075057 | *P. ornatus* phyllosoma, cultured | | Moderate |
| *Pseudoalteromonas* sp. (PT2) | PP81 | JX075058 | *P. ornatus* phyllosoma, wild | | Strong |
| *Pseudoalteromonas* sp. (PT2) | PP86 | JX075059 | *P. ornatus* phyllosoma, wild | | Strong |
| *Pseudoalteromonas* sp. (PT2) | PP87 | JX075060 | *P. ornatus* phyllosoma, wild | | Strong |
| *Ruegeria* sp. (PT1) | AH10 | JX075061 | Appendicularian (*Appendicularia*) | | Moderate |
| *Ruegeria* sp. (PT1) | K2 | JX075063 | Larviculture water (AIMS) | | Moderate |
| *Ruegeria* sp. (PT2) | EPP04 | JX075062 | *P. ornatus* phyllosoma, cultured | | Moderate |
| *Bacteroidetes* (PT1) | AH26 | JX075065 | Appendicularian (*Appendicularia*) | | Low |
| *Bacteroidetes* (PT1) | PPM04 | JX075064 | *P. ornatus* phyllosoma, cultured | | Low |

^a^Accession number in GenBank

^b^Based on inhibition zone in well diffusion assay: low (5-10 mm), moderate (11-20 mm) or strong (≥ 21 mm)

^c^All *Pseudoalteromonas* PT1 isolates showed yellow pigmentation on MMA

Abbreviations: PT: phylotype
