# Supplementary material for: Identification of an Antagonistic Probiotic Combination Protecting Ornate Spiny Lobster (Panulirus ornatus) Larvae against Vibrio owensii Infection
Source: PLoS One. 2012 Jul 5;7(7):e39667. doi: 10.1371/journal.pone.0039667 (PMC3390342; doi:10.1371/journal.pone.0039667)
Supplement: Protocol S1 — Monostrain biofilm production assay. (DOCX) [file pone.0039667.s003.docx]

**Protocol S1**

**Monostrain biofilm production**

MB was inoculated with PBS suspensions of *V. owensii* DY05, DY05[GFP] or candidate probionts (initial concentration 1 x 10^7^ CFU mL^-1^) in Nunc™ (NUN167008) microwell plates (final well volume 200 µL). Separate plates were used for each incubation period (12, 24, 36, 48, 72 and 96 h at 28^o^C) and treatments were carried out in hextuplicate. After incubation, wells were washed 3x in 200 µL PBS to remove planktonic and nonadherent cells. Two hundred microlitres of 0.4% CV was added to the wells and incubated at room temperature for 20 min. After staining, CV was removed and the wells were washed 3x in 200 µL PBS. The CV-stained biofilm was solubilised in 200 μL 95% ethanol and absorbance (OD_595nm_) was measured using a Wallac Victor2 1420 multilabel counter. Measurements were adjusted by subtracting background absorbance generated from a MB control.
